# Mid-upper arm circumference predicts the risk of gestational diabetes in early pregnancy

**DOI:** 10.1186/s12884-024-06664-z

**Published:** 2024-07-04

**Authors:** Ning Ma, Liwei Bai, Ziru Niu, Qiang Lu

**Affiliations:** 1 Department of Endocrinology, First Hospital of Qinhuangdao, Hebei, Qinhuangdao 066000 China; 2 Qinhuangdao Hospital for Maternal and Child Health, Hebei, Qinhuangdao 066000 China

**Keywords:** Mid-upper arm circumference, Gestational diabetes, Obesity, Predictive factor

## Abstract

**Background:**

The present work aimed to assess the value of mid-upper arm circumference (MUAC) at 8 to 12 weeks in predicting the occurrence of gestational diabetes mellitus (GDM).

**Methods:**

According to eligibility criteria, 328 women with singleton pregnancies who underwent routine antenatal check-ups at Qinhuangdao Maternal and Child Health Hospital from September 2017 to September 2020 were included. The patients were divided into the gestational diabetes mellitus (GDM) and non-GDM groups according to oral glucose tolerance test (OGTT) data from gestation weeks 24 to 28. Clinical data were compared between the two groups. Logistic regression analysis was performed to determine factors independently predicting GDM. Receiver operating characteristic (ROC) curve analysis was employed to analyze the value of MUAC in predicting the occurrence of GDM. The optimal cut-off points were calculated.

**Results:**

In logistic regression analysis, pre-pregnancy weight, waist circumference, MUAC, UA, TG, and HDL-C independently predicted the occurrence of GDM (*P* < 0.05). MUAC retained statistical significance upon adjustment for various confounders (OR = 8.851, 95%CI: 3.907–20.048; *P* < 0.001). ROC curve analysis revealed good diagnostic potential for MUAC in GDM (AUC = 0.742, 95%CI: 0.684–0.800, *P* < 0.001), with a cut-off of 28.5 cm, sensitivity and specificity were 61% and 77%, respectively.

**Conclusion:**

Pregnant women with MUAC >28.5 cm are prone to develop GDM during pregnancy, indicating that MUAC as an important predictive factor of GDM in early pregnancy.

## Introduction

Gestational diabetes mellitus (GDM) represents a major perinatal complication, whose prevalence is rising with the increasing number of overweight or obese women of childbearing age, with the current global pooled standardized prevalence of GDM being 14.0% [1]. GDM attracts increasing attention and focus for causing major adverse pregnancy outcomes and enhancing the risk of birth trauma, and hypoglycaemia in the immediate postpartum period, fetal macrosomia (excessive birth weight), shoulder dystocia, diabetes, hypertension, obesity, heart disease and other metabolic diseases in the offspring and the mother [2–4].The diagnosis of GDM is established after an oral glucose tolerance test (OGTT) at pregnancy weeks 24 to 28; however, even before this screening, pregnant women at elevated risk of GDM already show a trend of increased blood glucose, which is harmful for mother and child [5]. A previous study suggested that early detection and strict management of patients at high risk of GDM can improve maternal and infant outcomes [6]. Therefore, predicting the risk of GDM in early pregnancy and providing timely and targeted interventions to individuals at high risk of the disease may help improve maternal and infant outcomes.

Mid-upper arm circumference (MUAC) is a simple and easily obtained anthropometric parameter that was previously used to assess muscle mass and nutritional status or quality of life in humans [7–9]. A recent study found new applications for this old index [10]: MUAC is associated with overweight and obesity in young people [11], making it a simple tool to detect abdominal obesity and insulin resistance in diabetic individuals [12]. Currently, only a few studies have examined the correlation between MUAC and gestational diabetes, and it remains unclear whether MUAC could predict metabolic abnormalities and represent a new marker for predicting the occurrence of GDM.

## Materials and methods

### Study population

**Fig. 1:**
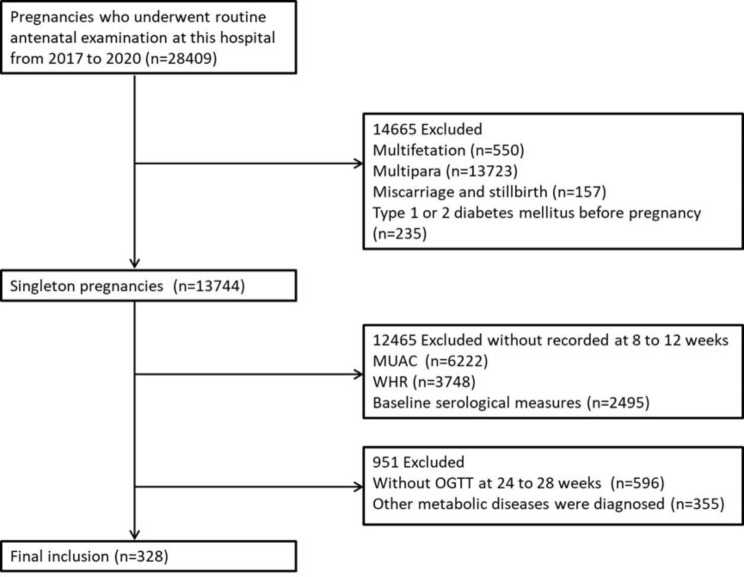
Study flowchart. MUAC: mid-upper arm circumference; WHR: waist-hip ratio; OGTT: oral glucose tolerance test

Pregnancies who underwent routine antenatal examinations in the Obstetrics Clinic of Qinhuangdao Maternal and Child Health Hospital between September 2017 and September 2020 were enrolled in this retrospective study. Figure 1 shows the detailed screening process for eligible participants. Inclusion criteria were singleton pregnancy, primipara and complete information about anthropometric measures and serum indexes. Exclusion criteria were diagnosis of diabetes mellitus prior to pregnancy, family history of diabetes, alcohol and smoking history, cancer, hypertension, cardiac disorders, thyroid disorders, and severe liver and kidney disorders. Ultimately, 328 women were included. This study had approval from the Ethics Committee of Qinhuangdao Maternal and Child Health Hospital and Qinhuangdao First Hospital.

All patients were examined by the 75 g OGTT at gestation weeks 24 to 28. GDM diagnosis followed the International Association of Diabetes and Pregnancy Study Groups (IADPSG) guidelines [13]: fasting plasma glucose (FPG) level ≥ 5.1 mmol/L, 1-h post-glucose (1hPG) level ≥ 10.0 mmol/L, or 2-h post-glucose (2hPG) level ≥ 8.5 mmol/L. Pregnant women with normal glucose levels were included as controls. The non-GDM (*n* = 225) and GDM (*n* = 103) groups were determined based on OGTT data.

### Measurements and definitions

#### General data collection

Maternal age, and pre-pregnancy weight (accuracy of 0.1 kg) and height (accuracy of 0.1 cm) were collected and recorded at the initial antenatal visit. Pre-pregnancy body mass index (BMI) was obtained as BMI = weight/height^2^ (kg/m^2^). Based on Chinese National Health Commission criteria, the patients were assigned to two weight groups: underweight and normal weight group, BMI < 24.0 kg/m^2^, overweight and obesity group, BMI ≥ 24.0 kg/m^2^ [14].

A pregnancy profile was generally created between 8 and 12 weeks of gestation. MUAC, waist circumference, and systolic and diastolic blood pressure levels were collected at this period. Measurements were completed by two trained examiners, with the participants standing relaxed with feet shoulder width apart and the arms drooped naturally. MUAC was measured on the right upper arm at the midpoint of the acromion and olecranon processes using a flexible tape. Waist circumference measurement was performed at the umbilical level after normal expiration. Hip circumference measurement was carried out at the most prominent part of the buttocks, accurate to 0.1 cm. Waist-to-hip ratio (WHR) was obtained as WC (cm)/HC (cm). In women, a WHR ≥ 0.85 was considered to indicate central obesity. Right brachial artery blood pressure was obtained with a mercury sphygmomanometer in the resting state. Blood pressure was measured twice and averaged for analysis.

### Laboratory tests

All participants underwent an 8–12 h fasting at night, and venous blood samples were collected in the next morning. An automatic biochemical analyzer (Hitachi 7060 type, Ibaraki, Japan) was employed for triglyceride (TG), serum total cholesterol (TC), high-density lipoprotein-cholesterol (HDL-C), low-density lipoprotein-cholesterol (LDL-C), alanine aminotransferase (ALT), aspartate aminotransferase (AST), gamma-glutamyl transpeptidase (GGT), and uric acid (UA) detection in early pregnancy (8 to 12 weeks), as well as plasma glucose at fasting, 1-h plasma glucose (1hPG), and 2-h plasma glucose (2hPG) in the second trimester (24 to 28 weeks). Analytical techniques included the continuous monitoring and two-point terminal methods. Regarding the accuracy of blood glucose detection, the coefficients of variation of intra-assay and inter-assay were less than 2% and 4.2%, respectively. The relative deviation between the measured and target values was ≤ ± 5%; blood glucose concentrations of 0.02 to 40.00 mmol/L were in the linear range (*r* ≥ 0.99), with an absolute deviation of ± 0.2 mmol/L.

### Statistical analysis

Data analysis used SPSS 25.0. To compare differences between the non-GDM and GDM groups, the two-sample t-test was used for continuous variates, described as mean ± standard deviation, while the chi-square (χ^2^) test was employed for categorical variates, represented as number and percentage. Binary logistic regression analysis was carried out to identify risk factors for GDM. Furthermore, independent variables that showed significant differences were divided into three tertiles, with the first tertile considered a reference for trend analysis. Odds ratios (ORs) and 95% confidence intervals (CIs) were determined. The association of MUAC with GDM was analyzed. The Crude Model had no adjustments. Age (Model 1), pre-pregnancy BMI, waist circumference, hip circumference, TG, UA, and HDL-C (Model 2) were adjusted. The area under the receiver operating characteristic (ROC) curve (AUC) was calculated to determine the diagnostic potential of MUAC for GDM, and the cut-off was obtained simultaneously. *P <* 0.05 indicated statistical significance.

## Results

### Clinical data

The 328 pregnant women were 21–40 years old, with an average age of 29.12 ± 4.55 years. Pre-pregnancy weight, BMI, MUAC, TG, UA, FPG, 1hPG, and 2hPG in 24 to 28 weeks were significantly higher in the GDM group compared with the non-GDM group, while HDL-C was significantly lower (all *P*<0.05). However, age, systolic and diastolic blood pressure levels, ALT, AST, GGT, TC, and LDL-C were similar in both groups (all *P*>0.05). Although waist circumference and hip circumference had significant differences (*P* < 0.001), central obesity classified by WHR had no significant difference (*P*>0.05) (Table 1).

**Table 1:** Clinical features [$\stackrel{-}{\varvec{x}}\pm \mathbf{s}$, n (%)]

Groups	non-GDM	GDM	t/χ^2^ value	*P*-value
Number of cases	225	103		
Age (years)	28.89 ± 4.52	29.63 ± 4.61	-1.365	0.173
Pre-pregnancy weight (kg)	58.98 ± 10.02	61.58 ± 10.16	-2.178	0.03
Pre-pregnancy BMI(kg/m^2^)			4.956	0.026
< 24.0 kg/m^2^	167(74.2)	64(62.1)		
≥ 24.0 kg/m^2^	58(25.8)	39(37.9)		
Pregnancy weeks 8 to 12				
Hips circumference (cm)	97.88 ± 8.34	101.35 ± 11.88	-2.675	0.008
Waist circumference (cm)	85.35 ± 8.72	89.61 ± 10.97	-3.779	<0.001
WHR			2.645	0.104
Normal	77(34.2)	26(25.2)		
Central obesity	148(65.8)	77(74.8)		
MUAC (cm)	27.19 ± 2.22	29.18 ± 2.40	-3.432	0.001
Systolic pressure (mmHg)	119.74 ± 11.0	118.71 ± 11.61	0.773	0.44
Diastolic pressure (mmHg)	72.53 ± 9.2	73.21 ± 10.06	-0.601	0.548
TG (mmol/L)	1.51 ± 0.59	1.92 ± 1.10	-3.583	<0.001
TC (mmol/L)	4.86 ± 0.92	4.70 ± 1.08	1.287	0.2
HDL-C (mmol/L)	2.06 ± 0.61	1.88 ± 0.55	2.536	0.012
LDL-C (mmol/L)	2.15 ± 0.68	2.03 ± 0.86	1.296	0.197
ALT (mmol/L)	17.58 ± 19.36	17.11 ± 16.79	0.215	0.830
AST (mmol/L)	18.11 ± 8.99	18.54 ± 7.60	-0.418	0.677
GGT (mmol/L)	11.90 ± 8.88	13.59 ± 9.75	-1.534	0.124
UA (mol/L)	193.33 ± 55.00	212.69 ± 61.85	-2.843	0.005
Pregnancy weeks 24 to 28				
0 h Plasma glucose (mmol/L)	4.54 ± 0.32	5.31 ± 0.46	-17.392	<0.001
1 h Plasma glucose (mmol/L)	7.11 ± 1.16	9.31 ± 1.83	-11.236	<0.001
2 h Plasma glucose (mmol/L)	6.07 ± 0.89	7.74 ± 1.84	-8.757	<0.001

GDM: gestational diabetes mellitus; BMI: body mass index. MUAC: mid-upper arm circumference; WHR: waist-hip ratio

### GDM risk factors

To analyze factors associated with GDM, univariate logistic regression analysis was performed. The analysis included maternal pre-pregnancy weight, pre-pregnancy BMI, MUAC, waist circumference, hip circumference, TG, UA, and HDL-C in early pregnancy, and FPG, 1hPG, and 2hPG in mid-pregnancy OGTT as independent variables. The results showed that waist circumference, hip circumference, UA, TG, and HDL-C independently predicted the development of GDM (all *P*<0.05, Table 2). Additionally, these independent variables were also presented for each tertile (Tertiles 1–3).

**Table 2:** Univariate logistic regression analysis of factors associated with GDM

Index	OR	95%CI	*P* value
Body weight	1.025	1.002–1.049	0.032
Tertile 1	1.0		
Tertile 2	1.387	0.768–2.504	0.278
Tertile 3	2.098	1.194–3.685	0.010
BMI	1.083	1.019–1.152	0.011
Tertile 1	1.0		
Tertile 2	2.278	1.245–4.169	0.008
Tertile 3	2.462	1.336–4.535	0.004
MUAC	1.480	1.307–1.675	<0.001
Tertile 1	1.0		
Tertile 2	2.696	1.491–4.873	0.001
Tertile 3	7.651	4.048–14.461	<0.001
Waist Circumference	1.048	1.021–1.075	<0.001
Tertile 1	1.0		
Tertile 2	2.220	1.217–4.049	0.009
Tertile 3	3.244	1.751–6.011	<0.001
Hip circumference	1.037	1.012–1.063	0.003
Tertile 1	1.0		
Tertile 2	0.857	0.481–1.529	0.602
Tertile 3	1.863	1.049–3.309	0.034
TG	1.911	1.381–2.644	<0.001
Tertile 1	1.0		
Tertile 2	1.0	0.545–1.834	1.000
Tertile 3	2.256	1.272–4.003	0.005
HDL-C	0.518	0.310–0.865	0.012
Tertile 1	1		
Tertile 2	0.410	0.231–0.728	0.002
Tertile 3	0.464	0.261–0.822	0.009
UA	1.006	1.002–1.010	0.006
Tertile 1	1		
Tertile 2	1.508	0.831–2.734	0.176
Tertile 3	2.056	1.144–3.693	0.016

Pre-pregnancy weight, pre-pregnancy BMI, MUAC, waist circumference, hip circumference, TG, UA, and HDL-C in early pregnancy, and FPG, 1hPG, and 2hPG in mid-pregnancy OGTT showed significant associations

### Association of MUAC with GDM

We further evaluated the association of MUAC with GDM. As a continuous variate, MUAC markedly contributed to the risk of GDM in all three models, with ORs of 1.480 (1.307–1.675), 1.476 (1.303–1.672), and 1.610(1.359–1.909), respectively. Consistently, MUAC as a categorical variable increased the risk of GDM with elevated categories from Tertiles 1 to 3. In the fully adjusted model (Model 2), the top tertile exhibited a 7.827-fold risk of GDM with the first tertile as a reference (Table 3).

**Table 3:** Logistic regression analysis of the relationship between MUAC and GDM in different models

Parameter	Crude Model OR (95%CI), *P*-value	Model 1 OR (95%CI), *P*-value	Model 2 OR (95%CI), *P*-value
MUAC	1.480(1.307–1.675)<0.001	1.476(1.303–1.672)<0.001	1.610(1.359–1.909)<0.001
Tertile 1	1.0	1.0	1.0
Tertile 2	2.696(1.491–4.873)0.001	2.611(1.428–4.774)0.002	3.004(1.557–5.797)0.001
Tertile 3	7.651(4.048–14.461)<0.001	7.514(3.963–14.246)<0.001	8.827(3.897–19.993)<0.001

Crude Model was adjusted for no index

Model 1 was adjusted for age

Model 2 was adjusted for age, pre-pregnancy BMI, waist circumference, hip circumference, TG, UA and HDL-C.

### Diagnostic potential of MUAC for predicting GDM

**Fig. 2:**
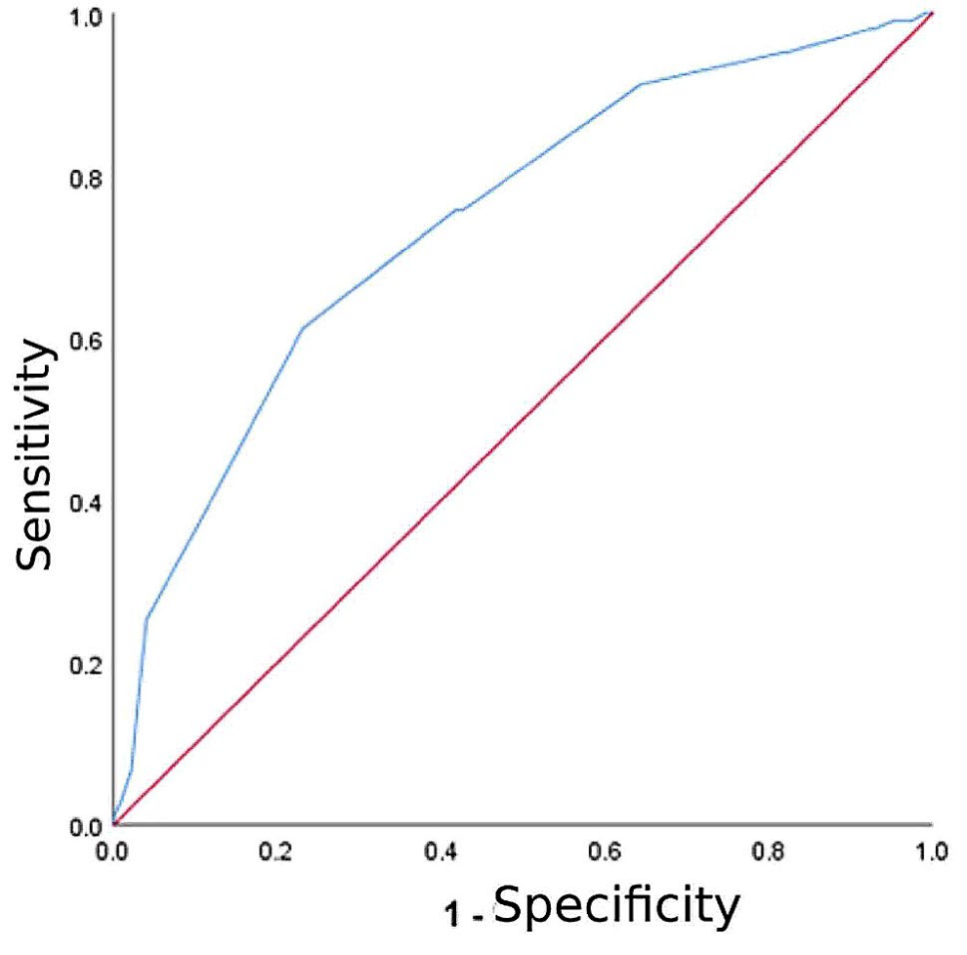
ROC curve analysis of MUAC for predicting gestational diabetes. AUC = 0.742 (95%CI: 0.684–0.800, *P* < 0.001), cut-off = 28.5 cm, sensitivity = 61% and specificity = 77%

ROC curve analysis was performed to determine the potential of MUAC to predict the occurrence of GDM. The cut-off value of MUAC for diagnosing GDM was 28.5 cm, and the associated AUC, sensitivity and specificity were 0.742 (95%CI: 0.684–0.800, *P* < 0.001), 61% and 77%, respectively (Fig. 2). AUC above 0.5 is considered to indicate good diagnostic potential.

## Discussion

The potential of MUAC as an early predictor of GDM in pregnant women was evaluated in the current work. The results showed a significant positive association between MUAC and GDM: for each 1-cm increment in MUAC, the risk of GDM was increased by 1.610-fold. Logistic regression analysis after adjustment for potential confounders and considering MUAC categories demonstrated that the relative risk of GDM in pregnant women in the top tertile of MUAC was 7.827-fold that of the first tertile. The AUC of MUAC for GDM prediction was 0.742, indicating that this risk factor correctly classified 74.2% of high-risk individuals. Therefore, MUAC as a potential factor for predicting GDM has a certain value.

Obesity and overweight were independently risk factors for GDM, the risk of GDM is increased almost 4-fold in women with obese and 9-fold in women with severely obese, compared to normal-weight pregnant women [15]. Excessive gestational weight gain prior to GDM screening test was a major risk factor for the development of GDM [16, 17]. Moreover, the association between GDM and pregnancy weight gain was mainly attributed to weight gain in early pregnancy [18]. However, GDM is diagnosed is at 24 to 28 weeks of gestation, that has lost the opportunity for early intervention. Thus, there are more studies required to explore predictive factors for early identification of GDM to facilitate targeted interventions in those most likely to benefit, this is of great significance to reduce the risk of GDM. BMI, waist circumference, and waist-to-hip ratio are commonly used to evaluate levels of obesity. These parameters changed significantly with the increase of uterine volume during pregnancy, and this affected the judgment of obesity or overweight in pregnancy. Previous studies demonstrated a significant association between maternal BMI and MUAC, MUAC can be used as a surrogate for BMI as it is measured easier and has less variability during the period of gestation unlike BMI [19]. Maternal MUAC is associated with the degree of obesity, body fat content, and the development of gestational diabetes [20]. A study on assessing 2912 pregnant women established a correlation between BMI and MUAC, the detection rate in overweight patients was 75% when MUAC ≥ 27 cm [21]. An UK research showed that the GDM prediction model combined maternal age, MUAC, systolic blood pressure, glucose, triglyceride, and HbA1c can well predict the risk of GDM in pregnant women [22]. Another study on the development of an early prediction tool for gestational diabetes employed MUAC, age, systolic blood pressure, HbA1c, and adiponectin reported a positive predictive value of 50% [23]. A random effect meta-analysis of 11 cohort studies showed that the risk of GDM was positively associated with maternal central obesity. There are many evaluation measures for maternal central obesity, such as waist circumference/waist-hip ratio, abdominal subcutaneous fat thickness and body fat index, but the predictive value of these measures is unclear [24]. In this study, the proportions of overweight and obesity in pregnant women were elevated in the GDM group compared with the non-GDM group, limited differences were detected (*P* = 0.026), but central obesity rate was no significant difference in both groups. Therefore, we speculated that high levels of MUAC in early pregnancy may independently predict the risk of GDM. However, MUAC in different races had different cutoff values in GDM prediction [25]. We determined an optimal cutoff of 28.5 cm, resulting in sensitivity and specificity of 61% and 77%, respectively, which indicated a medium predictive ability. Therefore, this study provided evidence for forecasting risk of GDM by MUAC in early pregnancy.

The Developmental Origin of Health and Disease (DOHaD) approach emphasizes that the intrauterine environment early in life has a significant impact on health and disease in adulthood [26]. GDM is considered to have an impact on placental development and function, the expression of parathyroid hormone-related protein (PTH-rP) and its receptor PTH-R1 in placenta are higher in GDM pregnant women with abnormal OGTT at fasting glucose compared to women with abnormal 60’ or 120’ glycemia, and the incidence of neonatal 1-minute Apgar score < 7 is higher in placental PTH-rP positive GDM women [27]. In pregnancies with maternal GDM, the placenta is exposed to environmental changes, such as increased inflammation and oxidative stress, dyslipidemia, and altered hormone levels, leading to abnormal fetal growth and development as well as metabolic and cardiovascular abnormalities in the offspring [28]. Many researches suggested that active perinatal management of pregnant women at elevated risk of GDM may reduce the incidence of GDM, which could further prevent or delay the development of long-term chronic diseases [29]. In a Finnish study, 293 pre-pregnant obese women and women in early pregnancy were randomized into the intervention and control groups. The intervention group underwent lifestyle interventions, and the results showed that moderate individualized lifestyle interventions decrease GDM occurrence in high-risk pregnant women by 39% [30]. In a Chinese randomized controlled trial of overweight and obese pregnant women, exercise interventions starting in early pregnancy significantly reduced GDM occurrence, with a 50% decline in the relative risk of GDM [31]. A meta-analysis [32] showed that pregnant women with GDM gained weight before 24 weeks of gestation, suggesting that interventions in overweight and obese pregnant women at elevated risk of GDM should be started at the earliest time, as starting interventions in mid- and late pregnancy does not exhibit a significant improvement in adverse pregnancy outcomes. This evidence suggested that early intervention is key to reducing the risk of GDM.

The establishment of early prediction models for GDM has attracted the common attention of many scholars. Different early and pre-pregnancy indicators are used to establish models to predict the risk of GDM. Sirico.et al observed that fetal heart rate (FHR) during the first trimester was associated with the development of pregestational diabetes mellitus and GDM [33, 34], and showed that FHR in the first trimester had a high predictive power for GDM. FHR is an indicator that must be monitored and easily obtained during prenatal examination, which is of great significance for prediction of GDM. This also suggests that we can capitalize on this indicator and combine with other parameters to improve the accuracy of GDM prediction in the future. Benevides.et al [35] identified the risk of GDM by using ultrasound abdominal fat measurement in early pregnancy and found that preperitoneal fat, rather than abdominal subcutaneous and visceral fat, could predict GDM, with an optimal cut-off point of 45.25 mm, a sensitivity of 73% and a specificity of 77% for predicting GDM. Obesity is closely related to the occurrence of GDM. Although abdominal fat ultrasound has a good predictive effect on GDM, abdominal ultrasound is not a routine examination item, and considering its time-consuming and patients’ wishes, it is not suitable for large-scale screening at present. Tenenbaum. et al. [36] developed an early prediction model for GDM by combining obesity, placental and inflammatory biomarkers, such as high BMI, insulin, sCD163, PP13, PAPP-A and TNFα. Combined indicators can improve the detection rate of GDM screening. However, the cost of special blood biomarkers is high and the large-scale application is limited, which is suitable for the in-depth study of the pathogenesis of GDM. Compared with other models, MUAC measurement can be widely used in the early diagnosis of GDM in terms of being simple, practical and cost effective.

In recent years, with overweight and obesity rates, the number of overweight and obese pregnant women has increased, resulting in a significantly increased risk of GDM. Insulin resistance is pronounced in overweight or obese women during pregnancy, thereby increasing the risk of developing GDM. MUAC is an old indicator with new applications but is simple and easy to use, with stable and reliable results and low variability. This and previous studies have further confirmed MUAC as a good indicator for clinical screening and prediction of GDM. It can efficiently assess the risk of developing GDM in a cost-effective manner and assist in the early detection of patients at risk of GDM. Thus, we should recognize the importance of early-life health and strengthen its management, which would reduce chronic diseases in adulthood and improve health across the lifespan.

There were limitations in this research. Firstly, the study was based on a small sample size, because of missing values in MUAC measurements for the pregnant women during the first trimester. And the impact of social and education level on the risk for GDM had not be considered. The pathophysiology of GDM involves β-cell dysfunction and insulin resistance during pregnancy but this study did not measure insulin; consequently, indicators relevant to MUAC and insulin resistance were not evaluated, and the mechanism was not discussed in depth. Despite these limitations, this study showed an association between MUAC and GDM in early pregnancy.

## Data Availability

All data generated or analysed during this study are included in this published article.
